# Identifying genes and traits associated with pre-eclampsia using summary statistics

**DOI:** 10.1371/journal.pone.0323683

**Published:** 2025-05-30

**Authors:** Wenyan Zhu, Qi Wang, Min Chen

**Affiliations:** 1 Department of Biostatistics, Center for Global Health, School of Public Health, Nanjing Medical University, Nanjing, China; 2 Department of gynecology and obstetrics, Nanjing Women and Children’s Healthcare Hospital, Women’s Hospital of Nanjing Medical University, Nanjing, China; University of North Carolina at Chapel Hill, UNITED STATES OF AMERICA

## Abstract

The occurrence and development of pre-eclampsia (PE) is closely related to genetics. However, multi-omics analysis does not provide sufficient evidence to define significant genes. Therefore, we aimed to identify significant genes and pathways using summary statistics from genome-wide association studies (GWAS). Based on the summary statistics, we used linkage disequilibrium score regression (LDSC) to discover genetic correlation between PE and complex traits. Leveraging summary statistics of tissue-specific expression quantitative trait loci (eQTL), we used FUSION to define significant genes, Bayesian colocalization analysis to identify pleiotropic genes, and Multi-marker Analysis of GenoMic Annotation (MAGMA) to determine the associated pathways. Specifically, considering the potential relationship between PE and tissues, we included 11 tissues, such as kidney cortex. Our integrative analysis revealed that the observed heritability of PE was 0.0179 (standard error [SE] = 0.0021, *P*-value < 0.001). Also, based on the Bonferroni correction, we defined 238 traits genetically correlated to PE, such as the other cardiovascular diseases (r = −0.55) and furosemide (r = 0.79). Integrating eQTL summary statistics across eleven tissues, we identified 30 significant genes, such as *EIF2S1* in the uterus (TWAS. Z = 4.44, TWAS. *P* = 8.95 × 10^−6^), and *PAWRP2* in ovary (TWAS. Z = 4.34, TWAS. *P* = 1.45 × 10^−5^). Based on colocalization, we identified 26 pleiotropic genes. We found that three genes, including *RPS26*, *SULT1A2*, *OBSCN-AS1*, and *SUOX*, were simultaneously defined by FUSION and colocalization. Moreover, we found that the significant enrichment was in the FOXG1_TARGET_GENES pathway regulated by the transcription factor *FOXG1* (P_FDR_ = 0.049). The findings of post-GWAS analysis for PE indicate that there are 30 significant genes and 26 pleiotropic genes. Future studies are required to investigate the efficacy of targeting pleiotropic genes to reduce the risk of PE.

## Introduction

Pre-eclampsia (PE) is a pregnancy-specific syndrome characterized by the onset of hypertension after 20 weeks of gestation, accompanied by at least one of the following complications: proteinuria, maternal organ dysfunction, or uteroplacental dysfunction [1]. Pre-eclampsia (PE) is a complex multisystem disorder associated with long-term health risks for both mothers and their offspring. Evidence indicates that women with a history of PE exhibit reduced life expectancy and an increased risk of developing stroke, cardiovascular disease, and diabetes. Furthermore, offspring born to mothers with PE may face a higher likelihood of cardiovascular and metabolic disorders in later life [2–4]. Previous epidemiological studies indicate that PE affects approximately 3–5% of pregnancies [5], and around 5–10% of pregnancies are affected by hypertensive disorders [6]. When PE begins 28 weeks of gestation, the risk of maternal mortality increases by 24 times compared to when it starts at term [7]. Without timely treatment, PE can lead to serious complications for both the mother and the infant, potentially threatening their lives [8].

From the patterns of onset for eclampsia and PE, increasing evidence suggests that genetics plays at least a partial role in its causality [9]. Advancements in high-throughput sequencing technologies have facilitated the identification of numerous genomic loci associated with pre-eclampsia (PE) through genome-wide association studies (GWAS). For example, a recent GWAS on PE identified 19 genome-wide significant loci, including 13 novel loci. Among these, seven loci contain genes previously implicated in blood pressure regulation, such as *NPPA*, *NPR3*, *PLCE1*, *TNS2*, *FURIN*, *RGL3*, and *PREX1* [10]. Another two-sample Mendelian randomization study indicated a strong causal relationship between rheumatoid arthritis (RA) and PE [11]. PE GWAS shows that most single nucleotide polymorphisms (SNPs) identified do not meet genome-wide significance thresholds, indicating that PE GWAS are still in their early stages [12].

Genetic correlations have emerged as crucial indicators for elucidating the shared genetic architecture of complex traits and the etiology of complex diseases. Estimating genetic correlations is a critical step in deciphering the shared genetic underpinnings of complex traits and diseases. Recently developed methods, such as Linkage Disequilibrium Score Regression (LDSC), have significantly advanced this field [13], Genetic and Environmental Covariance estimation by composite-likelihood Optimization (GECKO) [14], and Leveraging Allelic Variants Analysis (LAVA) [15] can compute global correlations between traits, while large-scale quantitative trait locus (QTL) data have been generated to determine the relationship between genotypes and gene expression (eQTL) [16,17]. This has facilitated the development of statistical techniques that integrate multidimensional data more easily. By contrast, transcriptome-wide association studies (TWASs) have been used to study the relationship between phenotypes and gene expression [18]. Furthermore, the emergence of methods, such as coloc [19] and SMR [20], enables us to investigate whether two traits share causal variants. Overall, integrating multidimensional eQTL data into GWAS data will help explore the pathogenesis of PE. Despite multiple attempts, there is no integration of multi-omics analyses based on GWAS, making it challenging to move from risk loci identification to therapeutic approaches.

Given the shortcomings of previous studies – such as the limited exploration of genetic correlations between PE and other complex traits, the lack of comprehensive multi-omics integration, and the reliance on European-centric data—this study proposes a series of post-GWAS analyses [21]. Specifically, we leverage the 1000 Genome Project (1000GP) as a reference panel for European individuals to ensure robust linkage disequilibrium (LD) estimation and SNP quality control. However, we acknowledge the need for future studies to include more diverse populations to enhance the generalizability of the findings. First, we estimated the heritability of pre-eclampsia (PE) and evaluated its genetic correlations with 4,347 other phenotypes from the UK Biobank (UKB). Next, by integrating expression quantitative trait loci (eQTL) summary statistics across 11 relevant tissues, we conducted transcriptome-wide association studies (TWAS) to identify associated genes and performed colocalization analysis to detect pleiotropic genes. Finally, we carried out enrichment analysis to explore the biological mechanisms underlying the significant genes.

## Methods

### GWAS summary statistics collection

We collected a PE summary statistic estimated by 296,824 participants in European (EUR) and Asian (ASA) from four cohorts, including 16,743 cases and 280,081 controls [10]. This PE summary statistic includes 14,447 European cases versus 278,022 European controls and 2,296 Asian cases versus 2,059 Asian controls. The ratio between case and control is 1:16.7, which might not be imbalanced. Data from the Finnish Genetics of Pre-eclampsia Consortium (FINNPEC, 1990–2011), the Finnish FinnGen project (1964–2019), the Estonian Biobank (1997–2019), and the previously published InterPregGen consortium [10]. This study included 12,556,951 SNPs [10]. Referring to the EUR individuals in 1000GP, we included SNPs: i) minor allele frequency (MAF) > 0.01; ii) Hardy Weinberg equilibrium (HWE)> 10E-7 [22–24]. After the stringent quality control for SNPs, we remained with 8,916,273 high-quality SNPs in the following analysis.

Additionally, for TWAS and genetic correlation analysis, we included the tissue-specific eQTL summary statistics and summary statistics for complex traits. For the eQTL, we also included eQTL data for 10 tissues with potential relationships with PE from FUSION (http://gusevlab.org/projects/fusion/), including Adipose-Subcutaneous, Adipose-Visceral, Adrenal Gland, Artery-Aorta, Artery-Coronary, Artery-Tibial, Heart-Atrial Appendage, Heart-Left Ventricle, Kidney-Cortex, and Uterus [20]. For the genetic correlation analysis, we included 4,347 phenotypes categorized under the ‘both_sexes’ category from the UK Biobank (UKB), excluding features marked as ‘pending’ in the md5 file. All of the summary statistics are shared freely with the scientific community on the Neale Lab website (https://nealelab.github.io/UKBB_ldsc/downloads.html/) [25].

### Genetic correlation

To examine whether PE and different genes in various tissues are influenced by common causal variants, we conducted colocalization analysis using the coloc method [19] on the summary data for PE and the eQTL data published for different tissues from GTEx [26]. For heritability estimation, LDSC regresses the LD score to the chi-square for each SNP. For genetic correlation, LDS regresses the product of marginal z -scores for the two traits on LD scores of each SNP. The majority of the GWAS data used included EUR Europeans; therefore, we regarded 503 EUR individuals as the reference panel. The coloc method is based on Bayesian statistics and calculates the posterior probability that there is a causal variant within a region affecting both traits by assigning priors to the association of each SNP within the region with the two traits. For the posterior probabilities calculated by coloc, we set a threshold of PP H4 > 0.95 to identify functional genes sharing genetic variants with the trait.

### Transcriptomics-wide association study

To explore functional genes associated with the trait in different tissues, we performed a transcriptome-wide association study (TWAS) using the Functional Summary-Based Imputation (FUSION) method [27]. This method estimates the association between predicted gene expression and a trait by calculating a weighted linear combination of SNP-trait standardized effect sizes, while accounting for linkage disequilibrium (LD) among SNPs. Using FUSION, we identified genes significantly associated with the trait across multiple tissues, and the Benjamini-Hochberg correction was applied to control the false discovery rate.

### Colocalization

Colocalization analysis is a popular technique for explaining how important signal sites impact the phenotype, which is required once GWAS analysis identifies significant signal sites [28]. We hypothesize that the loci identified by GWAS signals may modulate biological processes related to gene expression, thereby influencing phenotypic outcomes when eQTL colocalization and GWAS signals coincide. Colocalization analysis is commonly employed to determine whether two phenotypes are driven by the same genetic variant within a specific genomic region, thereby providing robust evidence for the previously observed association between the two phenotypes.

Specifically, when GWAS signaling and eQTL co-localization are detected, we assume that sites on the GWAS signaling may influence the phenotype by altering the biological processes of gene expression. SNP.PP H4 represents the posterior probability that the GWAS significant signal and eQTL site are the same site, ranging from 0 to 1. We treated genes with PP H4 > 0.95 as the pleiotropic genes. We used the *coloc* package (https://github.com/chr1swallace/coloc) to fit the colocalization model.

### Enrichment analysis

To investigate the biological function of pleiotropic genes and associated genes, we performed the enrichment analysis using Multi-marker Analysis of GenoMic Annotation (MAGMA), a tool for gene analysis and generalized gene-set analysis of GWAS data [29]. Based on multivariate regression models for gene and pathway analysis, it can be used to analyze both raw genotype data and summary statistics (https://ctg.cncr.nl/software/magma) [30]. Through MAGMA gene and gene set analysis, all relevant enrichment pathways containing the common genes found in TWAS and colocalization analysis were found, and significant pathways were selected by Benjamini-Hochberg correction. We used GWAS-associated genes to identify genes associated with specific traits or diseases from GWAS with the destination genome assembly of GRCh38/hg38.3. We conducted MAGMA analysis using the common genes defined by FUSION and colocalization in 11 tissues. We used the FUMA platform to provide functional enrichment results from MAGMA [25]. We set FDR < 0.05 as the threshold for the significant pathways.

## Results

### Genetic correlation between PE and complex traits

**Fig 1:**
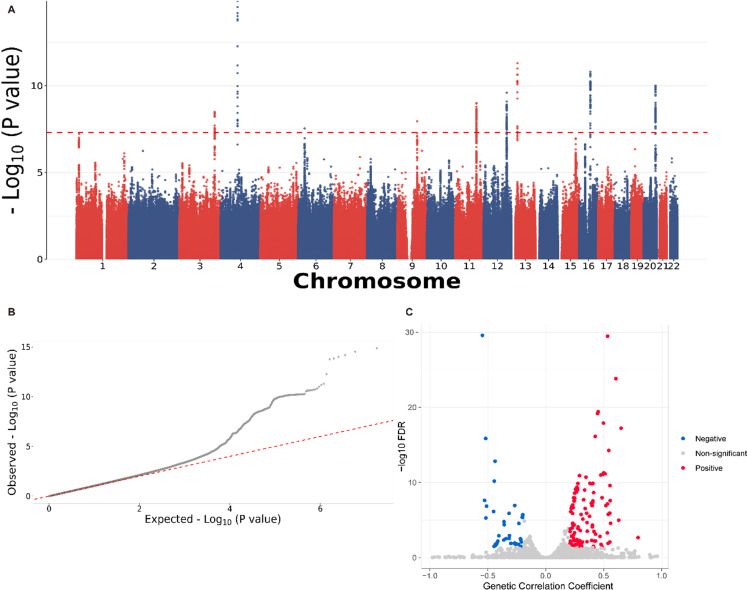
Summary for the GWAS analysis for PE. (A) Manhattan plot for PE. (B) Q-Q plot for PE. (C) Volcano plot for genetic correlation between PE and 4,347 complex traits.

Based on LDSC, the observed heritability of PE was 0.0179 (standard error [SE] = 0.0021, *P*-value < 0.001; Fig 1A). Consistent with the previous paper [31], we defined 289 significant SNPs with *P*-value < 1 × 10^−8^. The genetic inflation factor (*λ*) was 1.071, indicating that there may be a small number of SNP mutations (Fig 1B).

Additionally, we investigated the genetic correlation between PE and 4,347 complex traits with LDSC (Fig 1C). The observed heritability of 4,347 traits, on average, was 0.065 (ranging from −2.23, for Job SOC coding: paramedics, to 2.69, for recent medication for chronic bronchitis), in which the heritability of , e.g., fat percentage (left) traits was significant (FDR < 0.05). The 238 significant genetic correlations between PE and the complex traits ranged from −0.550 (*P*-value = 8.33 × 10^−34^, FDR = 2.65 × 10^−30^) for the other cardiovascular diseases to 0.7932 (*P*-value = 8.64 × 10^−5^, FDR = 2.16 × 10^−3^) for receiving diuretic therapy. For example, furosemide is a potent diuretic that primarily works by increasing urine excretion to reduce fluid volume in the body, thereby alleviating symptoms such as edema. For pregnant women with PE who have complications related to hypertension, doctors might consider using furosemide to manage fluid retention associated with PE [32].

### TWAS and colocalization genes and pleiotropic genes

**Fig 2:**
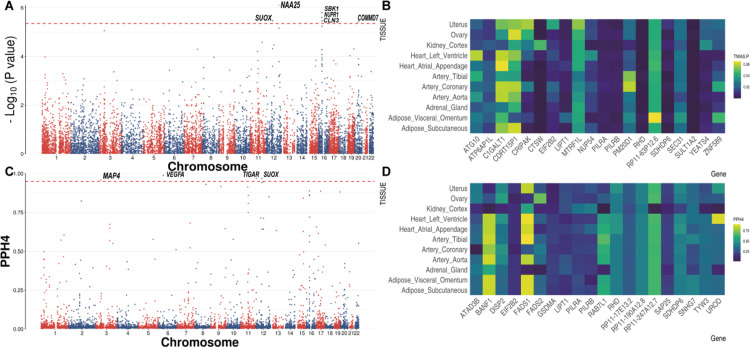
Summary for the TWAS and colocalization for PE. (A) Manhattan plot for TWAS. (B) Heatmap for the top 20 significant genes in TWAS. (C) Manhattan plot for colocalization. (D) Heatmap for the top 20 pleiotropic genes in colocalization.

We performed TWAS for PE by integrating with summary statistics of eleven human-related tissues using the FUSION pipeline. We identified 30 significant genes using Bonferroni correction (0.05/number of included genes). For thyroid, we defined 6 significant genes, the largest number of significant genes among the eleven tissues (Fig 2A, 2B and S1 Table). Six significant genes in thyroid were *SUOX* (TWAS. Z: 4.64, TWAS. *P*: 3.40 × 10^−6^), *NAA25* (TWAS. Z: −4.94, TWAS. *P*: 7.68 × 10^−7^), *SBK1* (TWAS. Z: −4.80, TWAS. *P*: 1.56 × 10^−6^), *CLN3* (TWAS. Z: 4.64, TWAS. *P*: 3.42 × 10^−6^); *NUPR1* (TWAS. Z: 4.71, TWAS. *P*: 2.49 × 10^−6^), and *COMMD7* (TWAS. Z: −4.62, TWAS. *P*: 3.77 × 10^−6^) (Table 1).

**Table 1:** Summary for significant genes in Thyroid with FUSION.

Gene	Chromosome	Position	Number of *cis-*SNP	Z	*P*-value
*SUOX*	12	55997179-55997180	293	4.64	3.40 × 10^−6^
*NAA25*	12	112109021-112109022	201	−4.94	7.68 × 10^−6^
*SBK1*	16	28292518-28292519	289	−4.80	1.56 × 10^−6^
*CLN3*	16	28495097-28495098	215	4.64	3.42 × 10^−6^
*NUPR1*	16	28539173-28539174	207	4.71	2.49 × 10^−6^
*COMMD7*	20	32743996-32743997	416	−4.62	3.77 × 10^−6^

1^st^ column: gene symbol; 2^nd^ column: chromosome; 3^rd^ column: chromosome location of the gene; 4^th^ column: the number of cis-SNP; 5^th^ column: Z-value; 6^th^ column: *P* value.

Moreover, we conducted fine-mapping on the TWAS result to identify causal variants for pleiotropic genes in relevant tissues, such as artery tibial and thyroid. Using SuSiE, we identified 10 genes with at least one causal SNP (*ALDH2, CDC37P2, CLN3, NUPR1, LINC00484, SUOX, NAA25, SBK1, CLN3, NUPR1*, and *COMMD7*). *CLN3* had 12 and 16 causal SNPs in artery tibial and thyroid, respectively (Table 2). The CLN3 gene is involved in basic physiological processes such as intracellular material transport and maintaining lysosomal homeostasis. In placental tissue, these cellular functions are crucial for the differentiation, proliferation of trophoblast cells, and the integrity of the placental barrier. In the placentas of patients with preeclampsia, abnormalities like insufficient invasion of trophoblast cells, placental ischemia, and hypoxia often occur [33].

**Table 2:** Summary for the fine-mapping for the associated genes.

Gene	CHR	Start	End	*P*(min)	Tissue	NO.SNP
*ALDH2*	12	111,766,886	111,766,887	3.18E-15	Artery Tibial	3
*CDC37P2*	16	28,413,702	28,413,703	3.49E-06	Artery Tibial	20
*CLN3*	16	28,495,097	28,495,098	6.02E-06	Artery Tibial	12
*NUPR1*	16	28,539,173	28,539,174	1.95E-41	Artery Tibial	11
*LINC00484*	9	91,159,572	91,159,573	9.67E-08	Artery Tibial	9
*SUOX*	12	55,997,179	55,997,180	1.91E-76	Thyroid	7
*SBK1*	16	28,292,518	28,292,519	1.42E-06	Thyroid	12
*CLN3*	16	28,495,097	28,495,098	1.54E-09	Thyroid	29
*NUPR1*	16	28,539,173	28,539,174	3.94E-08	Thyroid	19
*COMMD7*	20	32,743,996	32,743,997	2.26E-17	Thyroid	21

1^st^ column: gene symbol; 2^nd^ column: chromosome; 3^rd^ and 4^th^ columns: the start and end position for gene; 5^th^ column: minimum value for cis-SNP in summary statistics; 6th column: tissue; 7^th^ column: the number of causal SNP.

In addition, we performed COLOC for PE by integrating with summary statistics of human eleven related tissues. We identified 26 pleiotropic genes with PP H4 > 0.95 (Fig 2C and 2D and S2 Table). For artery tibial, we identified six pleiotropic genes with PP H4 > 0.95, including *SUOX* (PP H4 = 0.98), *AGRN* (PP H4 = 0.98), *CLCN6* (PP H4 = 0.97), *TIGAR* (PP H4 = 0.97), *GDNF* (PP H4 = 0.95), and *GDNF*-*AS1* (PP H4 = 0.97) (Table 3). Previous studies have identified separate loci of *CLCN6* associated with PE, with roles in natriuretic peptide signaling, angiogenesis, glomerular function, trophoblast development, and immune dysregulation [34]. For thyroid, we defined 4 significant genes, the largest number of significant genes among the eleven tissues (Fig 2B). The four significant genes in thyroid were *VEGFA* (PP H4 = 0.99), *TIGAR* (PP H4 = 0.97), *SUOX* (PP H4 = 0.97), and *MAP4* (PP H4 = 0.96) (Table 3).

**Table 3:** Summary of pleiotropic genes in Artery Tibial and Thyroid.

Gene	Tissue	Chromosome	Position	Lead SNP	PP H4
*SUOX*	Artery Tibial	12	56379427	rs1873914	0.98
*AGRN*	Artery Tibial	1	953678	rs113454255	0.98
*CLCN6*	Artery Tibial	1	11858957	rs45553335	0.97
*TIGAR*	Artery Tibial	12	4429331	rs191800458	0.97
*GDNF*	Artery Tibial	5	38060688	rs270594	0.95
*GDNF*-*AS1*	Artery Tibial	5	38056271	rs169187	0.97
*VEGFA*	Thyroid	6	43746169	rs3025000	0.99
*TIGAR*	Thyroid	12	4429331	rs191800458	0.97
*SUOX*	Thyroid	12	56393337	rs1081975	0.97
*MAP4*	Thyroid	3	48185266	rs9832957	0.96

1^st^ column: gene symbol; 2^nd^ column: tissues; 3^rd^ column: chromosome; 4^th^ column: chromosome location of the gene; 5^th^ column: lead SNP; 6^th^ column: PP H4 value.

**Fig 3:**
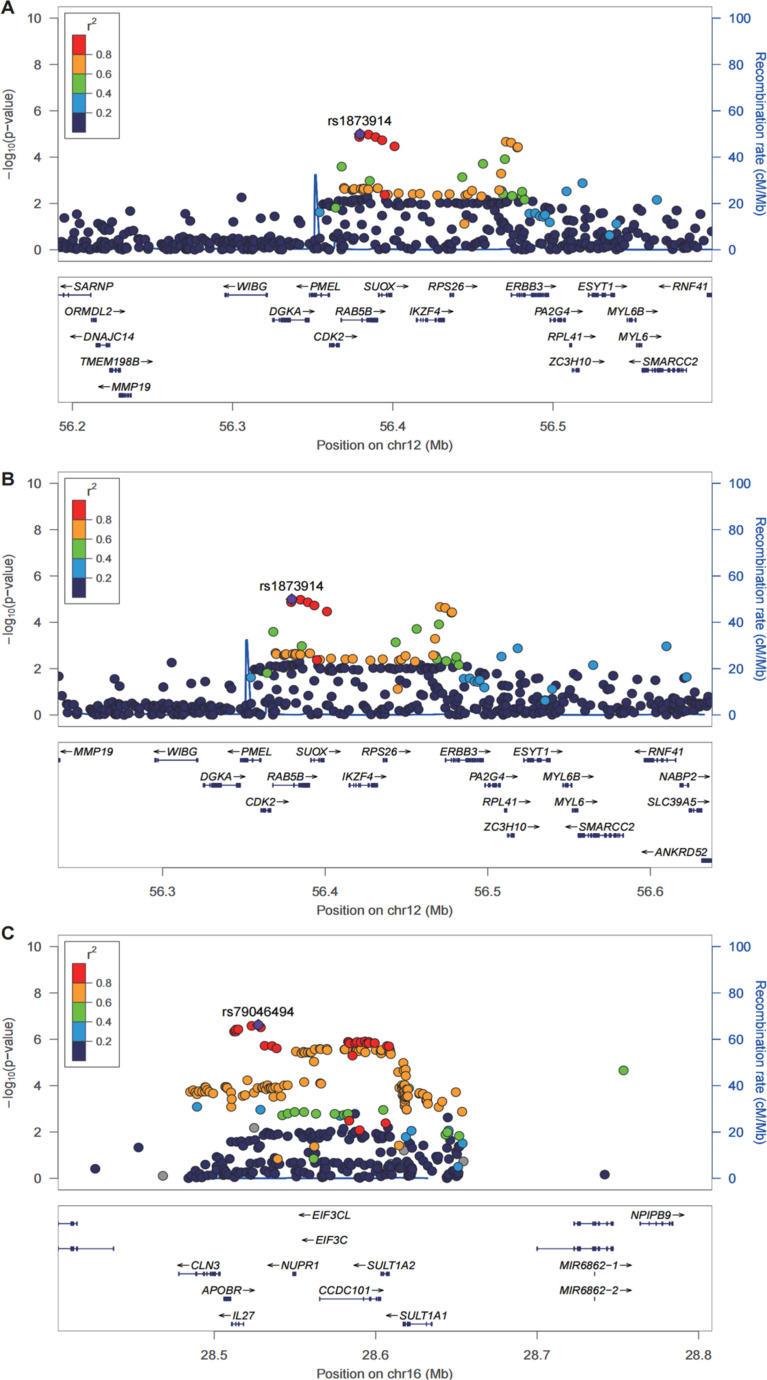
LocusZoom of *SUOX* *(A), RPS26* *(B)*, and*SULT1A2* (C) in the summary statistics of PE in thyroid.

We found that three genes, including *RPS26*, *SULT1A2*, and *SUOX*, were defined with TWAS and colocalization analysis (Fig 3). rs1873914 was the lead SNP for *SUOX* with PP H4 = 0.98, TWAS *P* = 3.40 × 10^−6^, and GWAS *P* = 1.01 × 10^−5^. Sulfite oxidase is a homomeric enzyme situated in the intermembrane space of mitochondria. *SUOX* changes in the activity or expression of sulfate metabolites may lead to changes that may have an impact on blood vessel function during pregnancy [35]. The rs1873914 was the lead SNP for *RPS26* with PP H4 = 0.96, TWAS *P* = 2.29 × 10^−5^, and GWAS *P* = 1.01 × 10^−5^. Vascular endothelial dysfunction in patients with PE is one of its typical manifestations. As a ribosomal protein, RPS26 primarily functions in protein synthesis; however, alterations in its expression might influence intracellular metabolic pathways, which could indirectly impact angiogenesis and endothelial function [36]. Finally, rs79046494 was the lead SNP for *SULT1A2* with PP H4 = 0.95, TWAS *P* = 4.77 × 10^−7^, and GWAS *P* = 2.37 × 10^−7^. *SULT1A2* is involved in hormone sulfation metabolism, especially estrogen metabolism. PE is associated with hormonal imbalances, so changes in *SULT1A2* activity may affect hormone levels and thus the development of PE [37].

### Enrichment analysis

**Fig 4:**
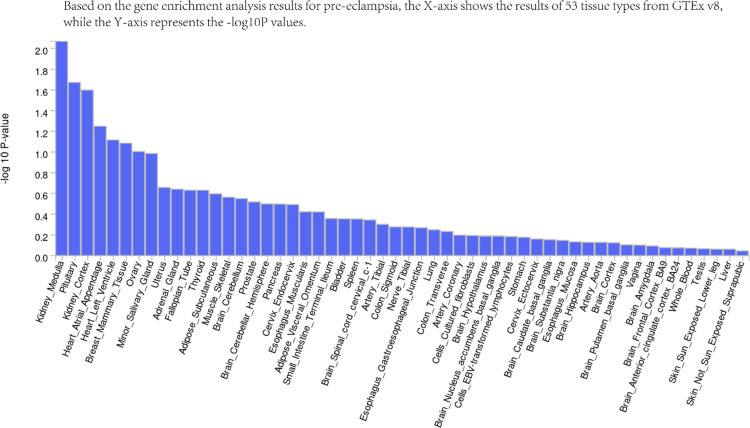
Summary for the results from MAGMA. *The x*-axis shows the pathway names, and *y*-axis shows the -log10 of the *P*-value. The kidney medulla displays the highest pathway enrichment score, suggesting its central role in PE pathogenesis. The pituitary gland and renal cortex also show significant enrichment, implicating their potential involvement in PE-associated gene regulatory mechanisms.

We used the summary statistics of PE to perform the enrichment analysis at the SNP level. Based on FDR, we obtained three significant pathways that have a role in the pathophysiology of PE, suggesting that the development and course of the illness may be significantly impacted by their dysregulation. Integrating the tissue information, the enrichment analysis from FUMA gives a more comprehensive picture for PE. The renal medulla and other organs might link to particular biochemical processes that facilitate the development of PE (P-value < 0.05) (Fig 4). It is possible that the genes expressed in the renal medulla are enriched in pathways associated with electrolyte balance and renal function, both of which are vital for preserving blood pressure equilibrium [38]. The expression of genes in the pituitary gland and renal cortex may also be enriched in pathways related to the regulation of hormones and renal filtration processes, respectively [39]. Moreover, according to MAGMA enrichment analysis, after correction for FDR of four genes, including *RPS26*, *SULT1A2*, *OBSCN-AS1*, and *SUOX*, were simultaneously defined by FUSION and colocalization, it was found that the significant enrichment was in a group of genes (FOXG1_TARGET_GENES) pathway regulated by transcription factor FOXG1 (BH_corrected_P_value = 0.049). There is *SULT1A2* in four genes on this pathway. Genes regulated by *FOXG1* may be involved in the function of vascular endothelial cells, thereby affecting vascular health during pregnancy.

## Discussion

In our study, we performed a comprehensive post-GWAS analysis for PE, including genetic correlation, TWAS, colocalization and enrichment analysis. In this post-GWAS analysis, we confirmed the genetic associations of PE. We used LDSC to estimate heritability, which is calculated by regression LD score and SNP chi-square statistics. In contrast, Steinthorsdottir et al. reported higher heritability estimates using Genomic Relatedness Restricted Maximum Likelihood (GREML) [40]. Different methods might cause different estimation for heritability. Our study examines the genetic correlations between 4,347 traits and PE, in contrast to the 894 pairs analyzed by Tyrmi et al [10]. Additionally, we employed MAGMA to perform enrichment analyses, whereas Tyrmi et al. focused on identifying associated genes. In their research, Tyrmi et al. conducted extensive data analyses and provided insights into the genetic architecture of PE [10]. We explored and identified 25,185 potential SNPs located on pathogenic loci that may influence PE. As a supplementary analysis, MAGMA was used, which is flexible and computationally fast, with greater power and less reliance on linkage disequilibrium data. It can directly use GWAS summary statistics as input data. The aim of using MAGMA was to demonstrate the relative differences in gene expression levels across different tissues, identifying the tissues where gene expression changes are most likely to contribute to the occurrence of PE. Integrating the tissue information, we observed significant associations for kidney medulla, pituitary, and kidney cortex (*P*-value < 0.05). In the past, Steinthorsdottir et al. reported the results of a meta-analysis of eight GWAS on preeclampsia in European and Central Asian women [40]. Based on a large meta-analysis, the study performed association analysis, estimated genetic correlation between 12 traits, and estimated the heritability using LDSC and GCTA. Based on summary statistics, our study examines the genetic correlations between 4,347 traits and PE and focuses on the post-GWAS analysis, such as colocalization to identify pleiotropy genes, TWAS to define significant genes, and enrichment analysis to identify relevant pathways [40].

PE is primarily caused by systemic endothelial dysfunction, which is characterized by reduced vascular integrity, endothelial damage, and impaired vasodilation [41,42]. Human studies support the potential importance of this heptapeptide during pregnancy, as an increase in urinary ANG-(1–7) has been observed throughout gestation. The initial rise in urinary ANG-(1–7) was detected at 12 weeks of gestation, but an increasing trend was observed as early as week 6 of pregnancy, suggesting that early adaptive changes in the renal RAS system may contribute to changes in fluid and electrolyte balance throughout pregnancy. Additionally, in normal late-term pregnant human subjects, plasma ANG-(1–7) levels increase, whereas in preeclamptic subjects, these levels decrease, indicating that a reduction in this peptide could be a significant factor in PE [43].This information can help guide subsequent experimental design and drug-targeted therapeutic strategies, e.g., interventions targeting tissues such as the renal medulla, ventricles, breast, thyroid, etc. may be useful in improving symptoms of PE.

This work revealed a common genetic etiology and possible pleiotropy between PE and thyroid by examining the genome-wide and localized genetic connections between the two conditions. In studies integrating and associating multi-omics data, it has been demonstrated that the expression of thyroid-related genes is associated with PE. Experiments have indicated that, compared to hyperthyroidism, subclinical hypothyroidism during pregnancy is associated with a higher risk of PE. TSH (Thyroid-Stimulating Hormone) exhibits a U-shaped relationship with PE. These findings quantify the risk of hypertension or PE in women with abnormal thyroid function tests, adding to the overall evidence of the risk of adverse maternal and fetal outcomes associated with thyroid dysfunction during pregnancy [44].

### Limitation

There are many restrictions on this study. First, the majority of the GWAS data used included Europeans, which limited the applicability of our findings to other ethnic groups. More varied groups should be included in future studies to confirm and build on these findings. Second, an exact causal relationship cannot be derived from this figure alone, as further experimental validation and other evidence are needed. Third, although we employ a variety of sensitivity analyses to address the issues of pleiotropy and heterogeneity, the inherent limitations of coloc and FUSION may still affect the robustness of our causal inferences. Traits with complex genetic architectures, such as those influenced by multiple small-effect SNPs, pose challenges for coloc. The presence of pleiotropy (where one SNP affects multiple traits) can complicate the interpretation of results. Both coloc and FUSION analyses assume that genetic factors are the primary drivers of observed associations. However, environmental factors can also play significant roles in modulating gene expression and disease risk, which may not be captured by these methods.

## Supporting information

S1 Table Summary for significant genes in ten tissues with FUSION. (XLSX)

S2 Table Summary for pleiotropic genes in ten tissues with coloc. (XLSX)
